# Water Splitting on Multifaceted SrTiO_3_ Nanocrystals: Calculations of Raman Vibrational Spectrum

**DOI:** 10.3390/ma15124233

**Published:** 2022-06-15

**Authors:** Veera Krasnenko, Leonid L. Rusevich, Aleksander Platonenko, Yuri A. Mastrikov, Maksim Sokolov, Eugene A. Kotomin

**Affiliations:** 1 Institute of Physics, University of Tartu, 50411 Tartu, Estonia; veera.krasnenko@ut.ee; 2 Institute of Solid State Physics, University of Latvia, LV-1586 Riga, Latvia; leonids.rusevics@cfi.lu.lv (L.L.R.); a.platonenko@cfi.lu.lv (A.P.); maksims.sokolovs@cfi.lu.lv (M.S.); jevgenijs.kotomins@cfi.lu.lv (E.A.K.); 3 Max Planck Institute for Solid State Research, 70569 Stuttgart, Germany

**Keywords:** STO, Raman calculation, DFT, stepped surface

## Abstract

Various photocatalysts are being currently studied with the aim of increasing the photocatalytic efficiency of water splitting for production of hydrogen as a fuel and oxygen as a medical gas. A noticeable increase of hydrogen production was found recently experimentally on the anisotropic faces (facets) of strontium titanate (SrTiO_3_, STO) nanoparticles. In order to identify optimal sites for water splitting, the first principles calculations of the Raman vibrational spectrum of the bulk and stepped (facet) surface of a thin STO film with adsorbed water derivatives were performed. According to our calculations, the Raman spectrum of a stepped STO surface differs from the bulk spectrum, which agrees with the experimental data. The characteristic vibrational frequencies for the chemisorption of water derivatives on the surface were identified. Moreover, it is also possible to distinguish between differently adsorbed hydrogen atoms of a split water molecule. Our approach helps to select the most efficient (size and shape) perovskite nanoparticles for efficient hydrogen/oxygen photocatalytic production.

## 1. Introduction

Thanks to the progress in the new materials development and understanding the dominating factors in the photochemical processes, great advancement has recently been made in the photocatalytic activity of various materials in visible light (e.g., [1]). Despite great achievements, many problems remain yet unsolved. For example, the efficient separation of photogenerated charges as well as catalytic reduction and oxidation sites on semiconductor-based photocatalysts remains one of the challenging issues in photocatalytic water splitting [2,3,4,5,6]. It can be noted that a low charge recombination rate is one of the key properties of a high-performance water splitting material. There are conflicting opinions about effect of nanoparticle size. For example, according to Townsend et al., the overall photocatalytic water splitting reaction is less effective at the nanoscale STO (with co-catalyst) particles due to increase of the water oxidation overpotential for the small particles (<30 nm) and a decrease in light absorption (due to a quantum size effect) [7]. However, Nuraje et al. consider that virus-templated STO nanowires have improved hydrogen evolution rate characteristics due to smaller particle size (∼5 nm in diameter) that provide a larger surface to volume ratio and the high crystallinity that prevents excited charge recombination at lattice defect sites [8]. The increase of the hydrogen production efficiency with STO facet nanoparticles was observed also by Mu [9] and Kržmanc [10]. As a result of recent developments in nanocrystal synthesis, materials with improved charge separation, achieved through heterojunction [10,11], mesocrystallinity [12], porosity [13] or exposed anisotropic facets [9,14,15,16,17]. The spatial separation of reduction and oxidation active sites can also become an effective method to reduce the recombination of photogenerated charge carriers that, in turn, can improve solar energy conversion efficiency (e.g., [18]).

According to recent developments, the photogenerated charge is shared between different facets of a semiconductor crystals with low symmetry [19,20]. Among other materials for water splitting, strontium titanate SrTiO_3_ (STO) is well known [15,18,21,22,23,24,25,26,27,28,29,30], where the processes of water adsorption and dissociation have been studied in detail [31,32,33]. Despite the fact that many semiconductor crystals are highly symmetric and have isotropic facets that are not suitable for charge separation between faces, it was found that the selective distribution on the anisotropic faces of 18-facet strontium titanate nanocrystals leads to a noticeable increase of apparent quantum efficiency [9]. Structurally, in 18-facet STO nanoparticles, the {0 0 1} facets coexist with the facets parallel to the {1 1 0} crystallographic plane. According to different models, an 18-facet nanoparticle is a natural platform for the efficient charge separation, whereas the reaction zone of the edges of a six-facet nanoparticle (which is a cube with the {0 0 1} faces) also differs from the {0 0 1} flat parts. A noticeable improvement of water splitting efficiency was also found experimentally on the anisotropic facets of STO nanocrystals. In the present study, we use an atomistic model of the {0 0 1} stepped surface, which is relevant to both six- as well as eighteen-facet STO nanoparticles. The challenging problem is identification of the most efficient facets and intermediate products of water splitting on nanoparticles which could be performed using the ESR (Electron Spin Resonance) and/or vibrational (Raman) spectroscopy [34,35].

To investigate the surface superstructure, direct observation of reproducible orbital selective tunneling on the STO (0 0 1) surface using scanning tunneling microscopy have been done by Song et al. [36]. In that observation, the electronic structures of the STO surface reversibly switched between different sets of symmetries depending on the bias of the sample. Such a switching was followed by a noticeable change in energy-dependent spectroscopy data. These observed experimental data were combined with density functional theory (DFT) calculations. As a result, it was clarified that symmetry breaking at the studied surface caused a crystal-splitting field of electron orbitals with strong in-plane anisotropy—this allowed the electrons alternately fill these orbitals during imaging with different biases. Therefore, understanding the atomic and orbital structure of the surfaces of complex oxides with broken symmetry is important to managing their functionality in new applications and opens up opportunities to explore novel physical and chemical properties.

Comparative ab initio calculations of HF and DFT using different exchange-correlation functionals and localized/plane wave basis showed the presence of crumpling and relative displacements of the second and third planes near the STO surface [37]. It was also found that structural transformations in low temperature tetragonal phase of STO associate with very tiny changes of the unit cell energy [37,38]. The group-theoretical analysis and first-principles hybrid DFT calculations have employed by Blokhin et al to prove the existence of a second-order anti-ferrodistortive phase transition in a defect-free ultrathin film of STO [39]. Low temperature structural transformations on the STO (0 0 1) surface of single crystals were studied theoretically and experimentally by Krainyukova et al [40], where structural anomalies were found depending on temperature. Obviously, the properties of the STO surface, including symmetry breaking, are also important for the study of effective water splitting.

Recent first principles study [41] of water splitting on stepped STO surface revealed very important results—three distinct adsorption regions of such the surface demonstrated very different catalytic activity. Thus, searching, validation and calculation of promising surfaces is a way to improve catalytic activity towards water splitting. In such a case, the calculations of Raman spectra of different anisotropic surfaces could help to distinguish the chemical adsorption of water derivatives on chosen surfaces and allow comparing calculated data with available experimental ones.

The calculation of Raman vibrational spectrum of chosen surface allows comparing the available experimental data of pure crystal and distinguishing the Raman frequencies, that are responsible for the specificity of the surface (size of the chosen nanostructure) as well as for chemical adsorption of water derivatives on surface. In order to estimate which Raman frequencies describe the stepped surface area, we performed a first-principles calculation of phonons and the Raman spectrum in the tetragonal phases of STO crystal and a thin film. As is well known, in the room temperature cubic phase of a perfect SrTiO_3_, no phonon modes are first-order Raman active and only a broad, multiphonon spectrum is observed [42]. However, the Raman peaks are still observed at room temperature due to lifting of inversion symmetry, caused by presence of defects, impurities or dopant ions, the grains or to the existence of strains (particularly in thin films). This is why in this study of water derivatives in SrTiO_3_ films we used SrTiO_3_ tetragonal phase.

## 2. Methods and Computational Details

**Figure 1:**
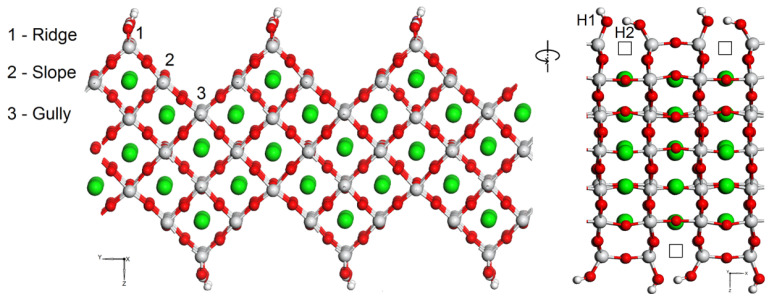
STO film with a faceted surface and with adsorbed water molecules at the “Ridge” site on both sides of the film (green, red, white, and gray balls are strontium, oxygen, hydrogen, and titanium atoms, respectively) [6]. The Sr-O bonds are not shown. The picture on the right is obtained by 90° rotation the left picture. The empty squares are oxygen vacancies. Hydrogen atoms originally belonged to water molecules adsorbed on the STO surface: H1 is a hydrogen atom still attached to the oxygen atom of the former water molecule, while H_2_ is the hydrogen atom that attached to the host oxygen atom removed from the surface (partial oxygen reduction).

The surface was designed and modeled as a thin STO film (slab) with steps, the slopes of which are parallel to {0 0 1} (see also ref. [41]). Although the surfaces {0 0 1} and {1 0 0} for a real strontium titanate material may not be equivalent depending on the temperature regime [43], such a distortion of the perovskite structure is irrelevant for the present study, and the surface orientations are given relative to the cubic phase. To cancel the dipole moment, the adsorbate was placed on both sides of the slab. Although the difference in the energies of the rhombohedral and tetragonal phases was insignificant, the bulk STO structure in the rhombohedral phase was taken as a matrix for the STO film with a stepped surface, since it turned out to be energetically more favorable. The difference in energy between distorted phases and cubic one is much larger than that between the different distorted phases. On such a stepped STO surface, there are three distinct areas of water adsorption—ridge, slope and gully (Figure 1). Among other areas of adsorption, the ridge one is of special interest because of oxygen vacancy formation upon water adsorption and their possible subsequent substitution/replacement by oxygen ions during the splitting of water molecules on this specific surface [41]. Thus, for the Raman calculations, a model with adsorbed derivatives of water on the ridge region of the STO stepped surface was chosen. To compare with the available experimental data and to estimate which Raman frequencies, describe the stepped surface area and adsorbates, we have also performed calculations of the Raman spectrum of the tetragonal phase of a bulk crystal.

**Figure A1:**
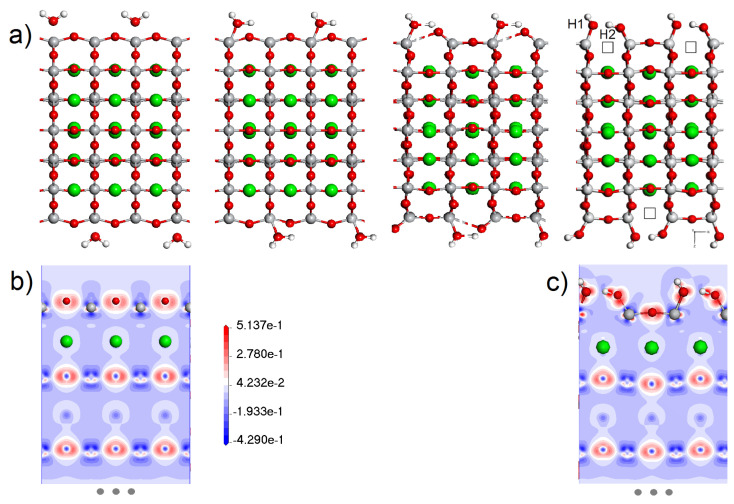
Formation of water derivatives (water splitting) on STO stepped surface: (**a**). schematic representation. The Sr-O bonds are omitted. The empty squares are oxygen vacancies. Isosurface of cross-section (charge) density difference (three atoms: titanium, oxygen and strontium near to surface) without (**b**) and with (**c**) adsorption of water derivatives.

Because of the stepped surface, oxygen atoms in the ridge region, participating in the creation of surface defects (oxygen vacancies), initially have only two bonds with the nearest titanium atoms (Figure A1, Appendix A). During the adsorption of a water molecule on a titanium atom at the surface, a redistribution of charge occurs between this titanium and the neighboring oxygen atoms of the STO surface, as well as between the atoms of the water molecule. In addition, there is a change in the charge of 4.3% of the strontium atoms closest to the created vacancy in comparison with the charges of strontium atoms in the slab without adsorbates. Charge redistribution leads to the breaking of the Ti-O bond with the simultaneous splitting of the water molecule. As the result, a second OH bond and an oxygen vacancy are formed on the STO film surface. Oxygen atoms “half detached” from the film surface form pure covalent bonds with titanium atoms, which are shorter and therefore more advantageous than Ti-O bonds in stepped STO film without adsorbates. Both water derivatives are adsorbed close enough to each other on the STO stepped surface, forming a small cluster of two OH groups supported by hydrogen bonding. Such a scenario (see also Figure A1) seems more plausible in comparison with the probability of the chemisorption of the hydrogen atom (H_2_) of the split water at a longer distance from the other part of the adsorbate (O-H1 group).

As the initial guess, the oxygen atom of the water molecule was placed in the immediate vicinity and directly atop the titanium atom in the region of the STO film ridge, and the O-H bonds of the water molecule were directed outward from the surface. The adsorbate concentration was one water molecule per 0.83 nm${}^{2}$ of the STO stepped surface.

The calculations were performed using the plane waves-based first principles method as implemented in the CASTEP module [44] of the Materials Studio package. The local density approximation (LDA) with the Ceperley–Alder–Perdew–Zunger (CA-PZ) functional [45] (pseudopotentials: norm conserving) was applied due to the low computational cost and energy consumption. The plane-wave basis energy cutoff was chosen as 750 eV for the STO bulk (10 atoms) and film with stepped surface (104 atoms + 6 atoms of two water molecules) models (the calculation of STO thin film with a flat {0 0 1} surface (26 atoms) was also performed). The calculated lattice parameters were a=b=5.410 Å, c=7.710 Å, and a=7.71 Å, b=10.92 Å, c=21.20 Å, α=β=γ=90° for bulk STO tetragonal phase and thin film with stepped surface, respectively: taking into account that a typical feature of such kind of calculations is that the LDA data are slightly underestimated if compared with the experimental values, the optimized structural constants for bulk STO tetragonal phase are in good agreement with experimental ones (a=b=5.507 Å, c=7.796 Å). The obtained angle of octahedral rotation is 2.03° that is very close to experimental value of 2.1° at 4.2 K [46]. Because the CASTEP calculations are periodic in all three directions, in order to exclude the interaction between the films in the calculations of the surface of STO thin films, a vacuum layer with thickness of 10 Å was used.

The Monkhorst–Pack scheme k-points grid sampling was set at 3×3×4 for the Brillouin zone for the bulk and 2×1×1 for the film model calculations. The convergence parameters were set as follows: total energy, $10^{-5}$ eV/atom; maximum force, 0.03 eV/Å; maximum stress 0.05 GPa; and maximum atomic displacement, $10^{-3}$ Å. The explicitly treated electronic configurations for the chemical elements forming the studied materials were as follows: $2s^{2}2p^{4}$ (O), $3s^{2}3p^{6}3d^{2}4s^{2}$ (Ti), $4s^{2}4p^{6}5s^{2}$ (Sr), $1s^{1}$ (H).

## 3. Main Results

### 3.1. The Bulk STO Crystal

**Figure 2:**
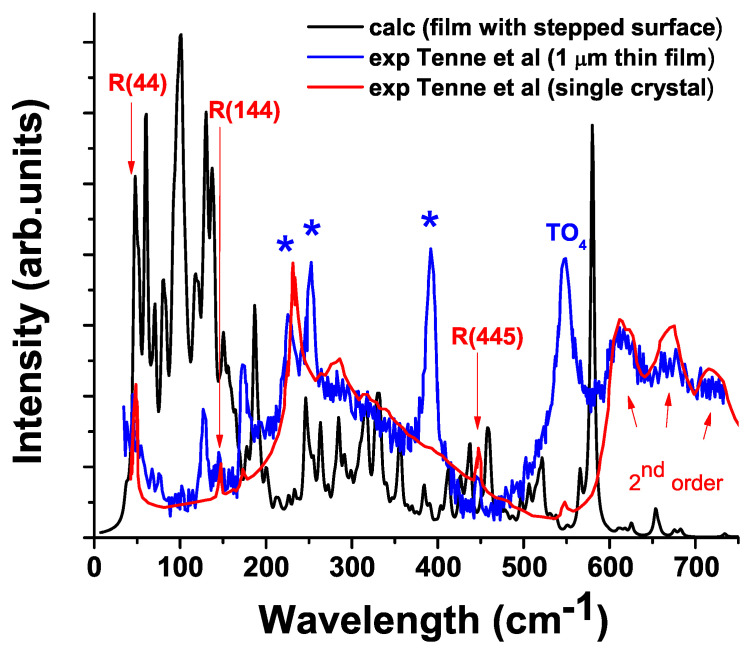
Raman spectra of calculated (black line, Lorentzian smearing is 5 cm${}^{-1}$) STO film with stepped surface, experimental [47] tetragonal STO single crystal and 1 μm STO thin film in the tetragonal phase (red and blue line, respectively). Structural modes from experimental data in bulk STO are marked with R (cm${}^{-1}$). Optical phonons from the SrRuO_3_ buffer layer are marked with stars: STO thin films grown on STO substrates with a SrRuO_3_ buffer layer [47,55,56]. The experimentally observed first order Raman scattering by hard-mode TO_4_ phonons are marked with “TO_4_” [47,55]. The temperature of 10 K and the incident light of 514.5 nm (Ar + laser line for excitation) were used for both calculation and experimental data.

**Figure A2:**
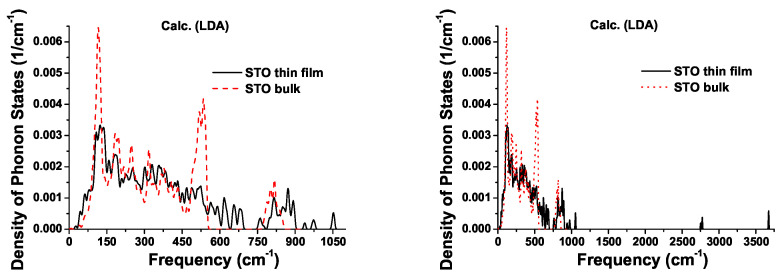
Density of phonon states of bulk STO crystal (red dashed line) and STO film with stepped surface (black line). Notice different frequency scales: normal (**right**) and enlarged for better visibility (**left**).

**Figure A3:**
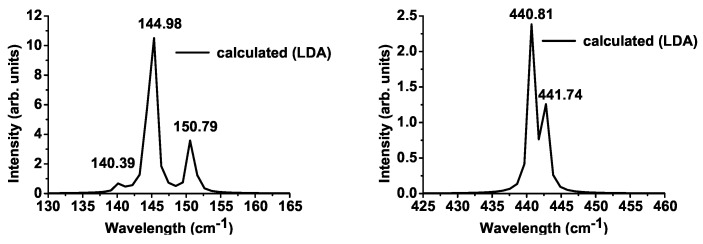
Raman spectrum of calculated bulk STO crystal: increased scaling for better visibility.

Both experimental and our computational studies have revealed that for pure STO crystals are 7 Raman active modes in the tetragonal phase with the I4/mcm space group. The calculated Raman-active frequencies of bulk STO crystal are in a good agreement with experimental data (see Table 1). These data are classified into IR-active and silent modes, Table A1 and Table A2 in Appendix A. In addition, the phonon density of states was calculated for a thin STO film and a bulk STO crystal (Figure A2, Appendix A). The most intensive peaks are at 45.23 cm${}^{-1}$ ($E_{g}$), 144.98 cm${}^{-1}$ ($E_{g}$), 150.79 cm${}^{-1}$ ($B_{2g}$), and 440.82 cm${}^{-1}$ ($B_{2g}$) (see Figure 2 and Figure A3 in Appendix A).

The $A_{1g}$ mode is shifted towards higher frequencies whereas $B_{2g}$ towards lower frequencies as compared with experimental data. According to previous calculations the closest to experimental value of $A_{1g}$ mode obtained by Evarestov et al. [38] using B3PW hybrid functional. The closest to experimental value of $B_{2g}$ mode obtained by Tenne et al. [47] using the density-functional perturbation theory (DFPT) approach, but the structural modes calculated using this approach are in a poorer agreement with experimental data—most likely due to the fact that the data obtained from phonon calculations performed for cubic phase of STO. To find out the reason for this discrepancy, we have carried out an additional calculation using the LDA and DS-VWN5 [48] functional implemented in CRYSTAL17 computer code [49]. As a result, the $A_{1g}$ value became closer to the experimental one. Obviously, the local density approximation does not have a sufficient theoretical level for such complex interactions, but it can still be improved by choosing more suitable functionals.

**Table 1:** Pure STO (SG 140, bulk), calculated and experimental Raman-active frequencies, cm${}^{-1}$.

	Our Calculations		Other Calculations							
Modes	LDA (CA-PZ)	LDA (DS-VWN5)	B1WC [50]	DFPT-LDA [47]	PBE [38]	PBE0 Opt [38]	B3PW [38]	PW PBE [38]	PW, LCAO [39]	Exp
$E_{g}$	45.23	39.85	21	59	48	79	76	17	17	15 [51], 40 [52], 11 [53]
$A_{1g}$	140.39	117.74	75	162	29	63	61	98	85	44 [47], 48 [51], 52 [52]
$E_{g}$	144.98	149.06	146	171	137	146	144	183	142	144 [47], 143 [51]
$B_{2g}$	150.79	154.48	148	230	152	158	157	140	157	235 [51], 229 [52], 224 [54]
$B_{2g}$	440.82	442.02	453.62	453	441	466	462	421		
$E_{g}$	442.75	444.51	454.2	557	444	468	465	425	454	445 [47], 460 [51], 447 [52], 420 [53]
$B_{1g}$	510.47	517.80	497	789	438	479	469	437		

**Figure A4:**
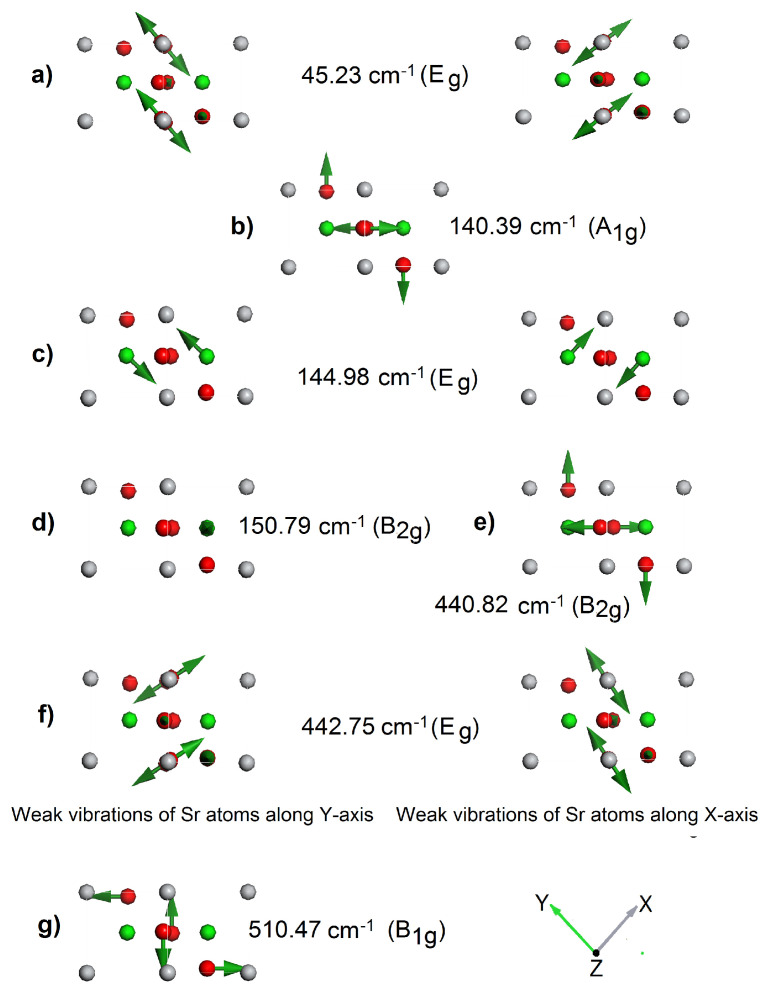
Mode representations for bulk STO in the tetragonal phase (Green—Strontium, Grey—Titanium, Red—Oxygen, calculated by LDA (CA-PZ)): (**a**) $E_{g}$ (45.23 cm${}^{-1}$), (**b**) $A_{1g}$ (140.39 cm${}^{-1}$), (**c**) $E_{g}$ (144.98 cm${}^{-1}$), (**d**) $B_{2g}$ (150.79 cm${}^{-1}$), (**e**) $B_{2g}$ (440.82 cm${}^{-1}$), (**f**) $E_{g}$ (442.75 cm${}^{-1}$) and (**g**) $B_{1g}$ (510.47 cm${}^{-1}$) The modes are visualized as vectors of vibration amplitudes, the visualization of weak vibrations are omitted.

According to our calculations (including additional ones), vibrations of Ti atoms do not participate in the Raman spectrum of STO. Our data are also consistent with those of Rusevich et al. [50] and Evarestov et al. [38]. Peaks $A_{1g}$, $B_{1g}$ and both $B_{2g}$ in the Raman spectrum are formed by rotating oxygen atoms that vibrate in the xy plane, whereas in the case of 150 cm${}^{-1}$ ($B_{2g}$) vibrations, the contribution of oxygen atoms is insignificant. Sr atoms vibrating along the tetragonal axis contribute to both $B_{2g}$ lines, although this contribution is significant for the 150 cm${}^{-1}$ line and negligible for the 440.82 cm${}^{-1}$ line. Oxygen atoms vibrating along and perpendicular to the z axis give the main contribution to the 45.23 cm${}^{-1}$ ($E_{g}$) line, while the contribution of Sr atoms vibrating in the xy plane is insignificant. The main contribution to the 144.98 cm${}^{-1}$ ($E_{g}$) line comes from the strontium atoms, which oscillate in the xy plane, the contribution of oxygen atoms is unnoticeable. For a better understanding, see Figure A4 in Appendix A, where the modes are visualized as vectors of vibration amplitudes.

### 3.2. STO Plane *{0 0 1}* Surface without and with Stepped Surface and Adsorbates

The calculated Raman spectrum of a thin STO film differs from the bulk spectrum, which also agrees with the experimental data (Figure 2) [47,54,55]. According to Hilt Tisinger et al. [56], when compared with bulk STO, the additional (Raman-forbidden) modes in STO film are induced by defects or lattice distortion in the films. In addition, the cations nonstoichiometry in homoepitaxial STO films also leads to the appearance of first-order peaks in the Raman spectra, which are symmetry-forbidden in the paraelectric STO [57].

Although structural R-modes should exist in the tetragonal phase, in their experiments Sirenko et al did not find R-modes of bulk STO (SG 140) in thin films (0.5 μm) [55]. As one of the possible reasons they cited the weaker intensity of R-modes compared to the Γ-point phonons, whereas the weakening of the intensity of the structural modes can occur due to the method of growing thin films, where the initially growing layers repeat the pattern of the substrate. Thus, the lattice mismatch between the film and the substrate acts as an external source of deformation by that lowering the symmetry of the film lattice [54,55,58]. In contrast to Sirenko et al. [55], Tenne et al. [47] have discovered first-order peaks of STO phonons in 1 μm films (as well as in reduced crystals), which indicates the existence of local regions with broken inversion symmetry, due to which the modes become Raman-active [47].

It was assumed that the symmetry of the thin films may be lower than the tetragonal structure due to strain, which is consistent with our calculations. As expected, the difference in structural data between the crystal and the film is enhanced near the surface. Due to the extremely small thickness of the modelled film, the difference is noticeable even in the centre of the film (see Appendix A, Table A3 and Table A4). Other factors that, in our case, cause differences in the properties of thin films from bulk ones, are oxygen vacancies, which are also common defects in thin oxide films [47,59,60]. All above mentioned factors affected the Raman spectrum.

The authors of previous experiments [61,62] suggest that the appearance of the broad bands at around 90 cm${}^{-1}$ is connected to the presence of defect-induced polar microregions. Additionally it was reported that dependence of the “soft” TO1 phonon frequency is different for STO thin films with different microstructural peculiarities [63]. It was also shown that the phonon dynamics in the low-frequency spectral region (10–100 cm${}^{-1}$) is also different for different STO nanocubes (average edge lengths of 60 and 120 nm) [54]. Apparently, in both cases (thin films and nanocubes), certain spectral features cannot be attributed the TO1 phonon [54]. The results of previous studies also show that these differences in the behavior of the band below 100 cm${}^{-1}$ arise due to polar domains, which occupy a much larger part of the total volume of STO nanocubes. Obviously, the low frequency region deserves further study since the “soft” phonon plays the important role in determining the dielectric properties of STO.

According to our calculations, the structural vibrational modes of a pure STO crystal differ from those of both a thin film with flat {0 0 1} and a stepped surface (Figure 2). In contrast to a pure crystal, where the structural modes have a special structure with certain vibrations of O or (and) Sr atoms (see Figure A4), and where the contribution of other atoms is negligible or none at all, the structural modes of the flat and stepped film do not exactly repeat the modes of the bulk STO crystal.

Moreover, the contribution of other atoms to the structural modes found to be more significant comparing to those in the bulk STO crystal. The Raman spectrum of a film with STO flat {0 0 1} surface, calculated taking into account symmetry, contains modes characterizing STO single crystal vibrations or close to them, as well as modes associated with surface vibrations. Compared to the calculated Raman spectrum without considering symmetry, the frequencies are slightly shifted (10 cm${}^{-1}$), and the vibrations with the highest amplitudes in both cases are mainly related to the vibrations of atoms on the surface of the STO film.

**Figure 3:**
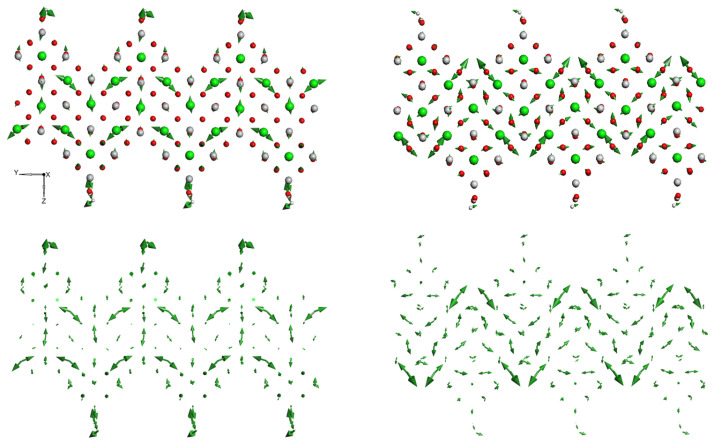
Examples of modes (image on the left −116.04 cm${}^{-1}$, on the right −150.35 cm${}^{-1}$) with specific patterns of STO film with stepped surface. Modes are visualized as vectors of vibrational amplitudes. The green, red, white, and gray balls are strontium, oxygen, hydrogen, and titanium atoms, respectively. The lower figures are given for a better view of the pattern of vibrational amplitudes of the stepped STO film: the atoms there are omitted.

We have also found that new features appear in the Raman spectrum due to vibrational modes associated with stepped specific film surface, defects and adsorbates (see Figure 2 and Figure 3). Some modes have a special pattern that allows one to identify the regions of a stepped surface (gully, slope and ridge). Some Raman frequencies that are responsible for the peculiarity of the stepped surface and specific vibrations of atoms in the pyramid (gully, slope and ridge regions) are shown in Figure 3 (see also Figure A6 in Appendix A).

**Figure 4:**
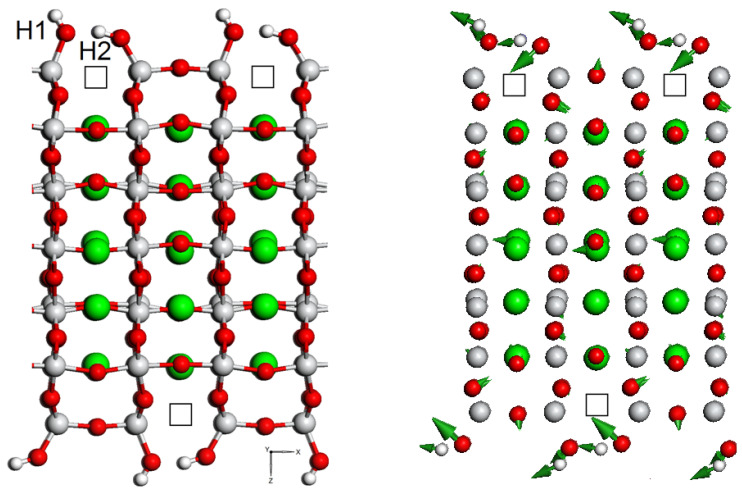
A faceted surface of STO film with adsorbed water molecules (left side, see also Figure 1) and representation of the mode (right side) of 102.15 cm${}^{-1}$, which may be related to the possibility of recovery the STO film surface from oxygen reduction. The green, red, white, and gray balls are strontium, oxygen, hydrogen, and titanium atoms, respectively. The Sr-O bonds are not shown. The empty squares are oxygen vacancies.

For a stepped surface with adsorbate, the calculated vibrational frequencies span from 7 to 3675 cm${}^{-1}$. There are almost no vibrations in the range of 1052–2751 cm${}^{-1}$. The peaks between 2751 and 3675 cm${}^{-1}$ belong to hydrogen vibrations: O-H2 modes have frequency of 2751–2753 cm${}^{-1}$, whereas O-H1 situated in 3674–3675 cm${}^{-1}$ range. The most important regions of the normal mode spectrum are below 150 cm${}^{-1}$, and around 580 cm${}^{-1}$ since these frequencies contribute the most to high amplitude fluctuations. For example, the mode of 102.15 cm${}^{-1}$ is associated with possibility to heal the adsorbate-induced partial oxygen reduction from STO surface (see Figure 4). According to our work, the frequencies at 658, 675, and 680 cm${}^{-1}$ correspond to the relative motion of Ti and O ions near the vacancy, whereas the highest frequency around 683 cm${}^{-1}$ mainly refers to the Ti-O stretching vibrations near the vacancy. According to Evarestov group [64], the frequencies of 505 and 630 cm${}^{-1}$ correspond to the relative motion of Ti and O ions near the vacancy, whereas the highest frequency around 700 cm${}^{-1}$ mainly refers to Ti-O stretching vibrations near the vacancy.

**Figure 5:**
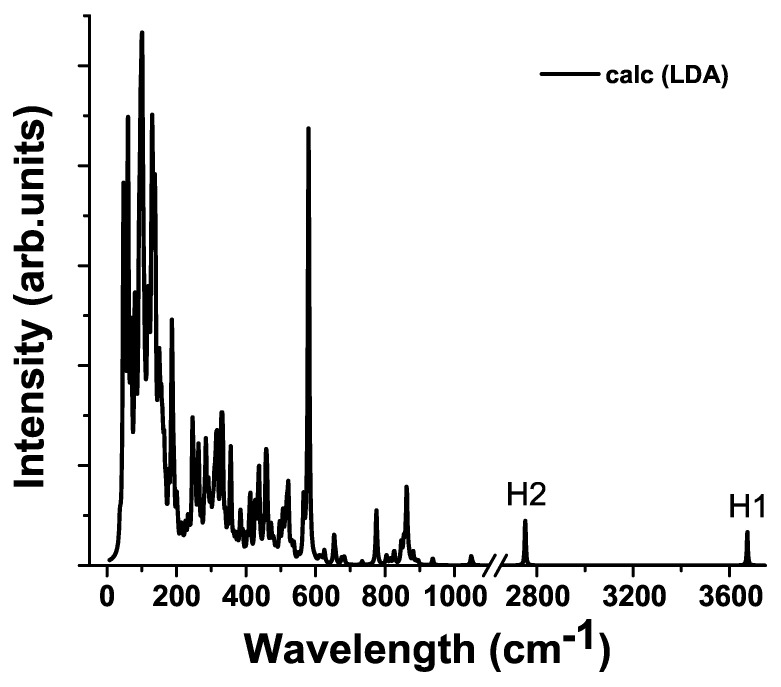
Raman spectrum of the STO film with stepped surface and with adsorbed derivatives of water molecules at the “Ridge” site on both sides of the film.

**Figure A5:**
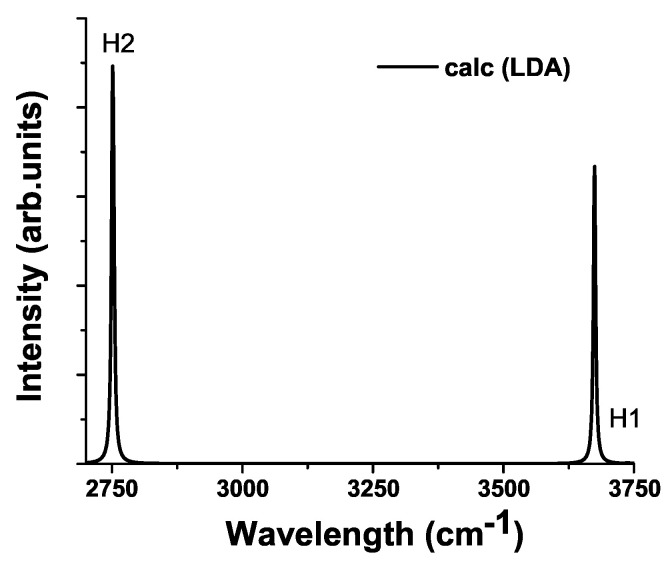
Raman spectrum of the STO film with stepped surface with adsorbed derivatives of water molecules (at the “Ridge” site on both sides of the film) in the range of 2700–3750 cm${}^{-1}$.

**Figure A6:**
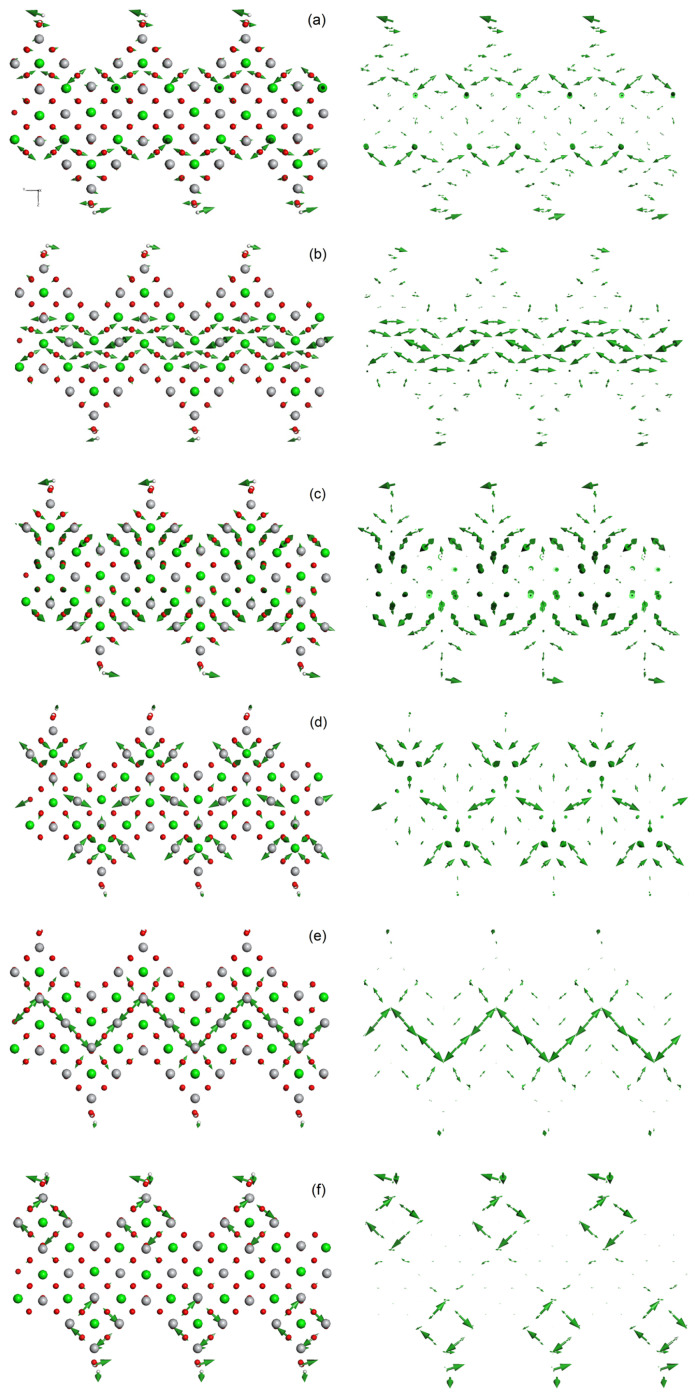
Examples of modes (image on the left—(**a**) 120.66 cm${}^{-1}$, (**c**) 246.72 cm${}^{-1}$, (**e**) 586.46 cm${}^{-1}$, on the right—(**b**) 162.61 cm${}^{-1}$, (**d**) 380.09 cm${}^{-1}$, (**f**) 657.90 cm${}^{-1}$) with specific patterns of STO film with stepped surface, calculated by LDA (CA-PZ). Modes are visualized as vectors of vibrational amplitudes. The green, red, white, and gray balls are strontium, oxygen, hydrogen, and titanium atoms, respectively. Right hand side figures are given for a better view of the pattern of vibrational amplitudes of the stepped STO film: the atoms are omitted.

In addition, the calculated Raman spectrum of stepped STO film makes it possible to distinguish the Raman frequencies responsible for the chemical adsorption of water derivatives on the surface (Figure 5, (Figure A5 Appendix A)).

Our simulation indicates that the hydrogen atom H1 of O−H group chemisorbed onto Ti atom of STO surface exhibits stretching vibrational frequencies of 3674 and 3675 cm${}^{-1}$. This is consistent with typical OH stretching band at 3100–3700 cm${}^{-1}$ (see e.g., Ref. [65] (3100–3690 cm${}^{-1}$ in solid hydroxides); Ref. [66] (3400–3800 cm${}^{-1}$ in fluor-elbaite); Ref. [67] (2900–3800 cm${}^{-1}$ in water)). On the contrary, the hydrogen atom H2, chemisorbed on the oxygen atom displaced from the surface, exhibits stretching vibration frequencies of 2751 and 2753 cm${}^{-1}$. The decrease of the vibrational frequency has been interpreted to be caused by hydrogen bond formed between formed OH groups as well as by increasing of the metal oxygen bond strength [33]. It is known fact that the formation of a hydrogen bond leads to significant changes in the Raman spectra.

Depending on hydrogen bond length and angle frequency shifts can reach the order of hundreds of cm${}^{-1}$. The straighter and shorter hydrogen bond has a lower vibration frequency. The formed covalent Ti-O bonds of Ti-O-H2 and Ti-O-H1 have distances of 1.86 and 1.75 Å, respectively. It should be noted that formed lengths are shorter than that of 1.93 Å in a pure STO crystal indicating benefit of the covalent bond formation.

According to our calculations the length of hydrogen bond (*d*(O-O)) is 2.48 Å and the angle (∡O-H-O) of H-bond bent is 162.3°. Remembering that LDA calculations typically underestimate experimental lattice constants, the calculated H-bond frequencies are in good agreement with the correlation of O-H stretching frequencies and O-H-O hydrogen bond lengths in minerals and in a good agreement with available experimental (see [68] and references therein) as well as theoretical data [33].

Thus it was found that it is possible to discern one of the hydrogen atoms that originally belonged to the water molecule from the other one: a hydrogen atom (H1) attached to the oxygen atom of the former water molecule vibrates at higher frequencies than hydrogen atom (H2) attached to the STO oxygen atom (Figure 1 and Figure 5 and Figure A5 in Appendix A). In case of stepped STO surface the close position of water derivatives on the STO surface leads to OH induced cluster formation, where the hydrogen bridges caused lowering of the hydrogen stretching vibrations in O-H groups.

## 4. Conclusions

Despite known limitations of the local density approximation (LDA), the calculated Raman spectra are in a good agreement with other theoretical calculations (including those with hybrid functionals), while the computational costs are noticeably much lower. It should be noted that proper management of available computational resources and reasonable energy consumption are extremely important nowadays, so choosing to use less calculation time with reasonable results contributes to this goal.

The calculated Raman-active frequencies of the slab and bulk STO crystal are in good agreement with the experimental data. The results of the Raman calculation of stepped STO surface with adsorbed water derivatives are also comparable with available experimental data. The calculated Raman spectrum of a thin STO film differs from the bulk spectrum, which also agrees with the experimental data. Moreover the calculated Raman spectrum of stepped STO film allows to identify the specific Raman frequencies for chemical adsorption of water derivatives (OH) on such a facet surface. In particular, it is possible to distinguish one of the hydrogen atoms that originally came from the water molecule from another one: a hydrogen atom (H1) attached to the oxygen atom of the former water molecule vibrates at higher frequencies than hydrogen atom (H2) attached to the oxygen removed from the surface.

We also have found that new features appear in the Raman spectra due to vibrational modes associated with the stepped film surface (Figure 2 and Figure 3). The water adsorption in the ridge position is accompanied by a pronounced structural reconstruction—breaking of the Ti-O bonds. In spite of this, the other two distinct surface regions of water adsorbtion (slope and gully) could also be important for further Raman calculations. It was shown that defects caused by water adsorption give the characteristic Raman frequencies that can be used to identify adsorbates and facet surfaces promising for photocatalysis. This approach opens the way to selection of the most catalytically efficient perovskite nanoparticles for water splitting and hydrogen production.

## Appendix A

**Table A1:** Pure STO (SG 140, bulk), calculated and experimental IR-active frequencies (cm${}^{-1}$).

	IR (Pure STO 140)			
	Bulk, Calc			
	Other Calc [50]	Our Calc		
Mode Rep	B1WC, Crystal	LDA (CA-PZ), Castep	LDA, Crystal	Exp [52]
Eu	47	66.05	115.48	15
A2u	53	75.34	126.77	
Eu	173	173.14	181.38	172
A2u	175	180.02	184.63	
Eu	260	250.51	251.06	
Eu	453.64	441.75	450.84	436
Eu	554	544.99	560.54	548
A2u	556	548.83	564.01	

**Table A2:** Pure STO (SG 140, bulk), calculated silent frequencies, cm${}^{-1}$.

	IR (Pure STO 140)		
	Bulk, Calc		
	Other Calc [50]	Our Calc	
Mode Rep	B1WC, Crystal	LDA (CA-PZ), Castep	LDA, Crystal
B1u	263	(B2u) 264.78 (O)	260.97
A1u	453	437.02 (Ti)	447.64
A2g	495	498.90 (O)	511.36
A2g	872	853.50 (O) Octa	860.34

**Table A3:** The difference of structural data of STO crystal and film of strontium titanate with plane {0 0 1}.

	Distances in Å			
	Slab	Bulk	Slab	Bulk
	Sr-Sr		Sr-O	
SrO layer	4.09—between SrO layers 3.79—in SrO layer	3.86—between SrO layers 3.83—in SrO layer	2.62, 2.97—between SrO and neighbouring central TiO_2_ layer 2.60, 2.64—between SrO and neighbouring surface TiO_2_ layer 2.68—in SrO layer	2.58, 2.86—between SrO and neighbouring TiO_2_ layer 2.71—in SrO layer
	Ti-Ti		Ti-O	
TiO_2_ layer	3.77—between TiO_2_ layers 3.79—in TiO_2_ layers	3.86—between TiO_2_ layer 3.83—in TiO_2_ layer	1.94 0 between central (TiO_2_) and neighboring SrO layer 1.83—between surface (TiO_2_) and neighboring SrO layer 1.90—in the surface (TiO_2_) layer 1.91—in the central (TiO_2_) layer (in the center of the film)	1.93—between TiO_2_ layers 1.92—in TiO_2_ layer

**Table A4:** The difference of structural data of STO crystal and film of strontium titanate with stepped surface.

	Distances in Å			
	Sr-Sr		Sr-O	
	Stepped Slab	Bulk	Stepped Slab	Bulk
SrO layer	3.66–3.79—gully 3.80–3.85—ridge (depending on the directions) 3.65–4.03—between SrO layers 3.79—in central SrO layer	3.86—between SrO layers 3.83—in SrO layer	2.60–2.92—gully 2.51—2.89 and 2.54–2.99—ridge (H atom towards and outwards to nearest slope, respectively)	2.58, 2.86—between SrO and neighboring TiO_2_ layers 2.71—in SrO layer
	Ti-Ti		Ti-O	
TiO_2_ layer	3.69—gully 3.54–3.72—ridge 3.75—in the center of the film	3.86—between TiO_2_ layers 3.83—in TiO_2_ layer	1.86–1.92—gully 1.90–1.99 and 1.87–1.99—slope (H atom towards and outwards to nearest slope, respectively) 1.78–1.80—ridge 1.91–1.95—in the center of the film	1.93—between TiO_2_ layers 1.92—in TiO_2_ layer
